# CRISPR Loss-of-Function Screen Identifies the Hippo Signaling Pathway as the Mediator of Regorafenib Efficacy in Hepatocellular Carcinoma

**DOI:** 10.3390/cancers11091362

**Published:** 2019-09-13

**Authors:** Shigeki Suemura, Takahiro Kodama, Yuta Myojin, Ryoko Yamada, Minoru Shigekawa, Hayato Hikita, Ryotaro Sakamori, Tomohide Tatsumi, Tetsuo Takehara

**Affiliations:** Department of Gastroenterology and Hepatology, Graduate School of Medicine, Osaka University, 2-2 Yamadaoka, Suita, Osaka 5650871, Japan

**Keywords:** CRISPR, pooled library, Hippo signaling pathway, HCC, regorafenib

## Abstract

Regorafenib is used for hepatocellular carcinoma (HCC), but its response does not last long, partly due to chemoresistance acquisition. We performed a clustered regularly interspaced short palindromic repeats (CRISPR)-based loss-of-function genetic screen and aimed to discover molecules involved in regorafenib resistance in HCC. Xenograft tumors established from Cas9-expressing HCC cells with pooled CRISPR kinome libraries were treated with regorafenib or a vehicle. Sequencing analysis identified 31 genes, with the abundance of multiple guide RNAs increased in regorafenib-treated tumors compared to that in vehicle-treated tumors, including 2 paralogues, LATS2 and LATS1, core components of the Hippo signaling pathway. Notably, all eight designed guide RNAs targeting LATS2 increased in regorafenib-treated tumors, suggesting that LATS2 deletion confers regorafenib resistance in HCC cells. LATS2 knockdown significantly mitigated the cytotoxic and proapoptotic effects of regorafenib on HCC cells. LATS2 inhibition stabilized the Hippo signaling mediator YAP, leading to the upregulation of antiapoptotic Bcl-xL and the multidrug resistance transporter ABCB1. Among 12 hepatoma cell lines, the half maximal inhibitory concentration (IC50) values of regorafenib were positively correlated with any of YAP, Bcl-xL and ABCB1 levels. The inhibition of YAP or Bcl-xL in regorafenib-insensitive HCC cells restored their susceptibility to regorafenib. In conclusion, our screen identified the Hippo signaling pathway as the mediator of regorafenib efficacy in HCC.

## 1. Introduction

Hepatocellular carcinoma (HCC) is the primary histological type of liver cancer and the most common cause of cancer-related deaths, especially in Asia and Africa [1]. Surgical resection or transplantation are the curative therapeutic options, but they can be applied to only 10 to 20% of HCC patients because of donor shortage, liver dysfunction or detection at an advanced tumor stage [1]. Furthermore, even after curative resection, HCC often recurs at a very high rate, since the liver is already predisposed to tumor development due to sustained chronic damage caused by viral infection, alcohol, lipids, etc. For unresectable or recurrent advanced HCC, therapeutic options are very limited, and sorafenib has been the only Food and Drug Administration (FDA)-approved drug for approximately a decade. However, the RESORCE (regorafenib for patients with hepatocellular carcinoma who progressed on sorafenib treatment) trial, a randomized, double-blind, placebo-controlled, phase 3 trial of regorafenib, was recently conducted for patients with HCC who progressed on sorafenib treatment and showed statistically significant improvement of overall survival compared to patients who received the placebo. Since 2017, regorafenib can be used for advanced HCC as the second line of therapy after the failure of sorafenib treatment [2,3].

Regorafenib is a multiple tyrosine kinase inhibitor that blocks the activity of a variety of kinases, including VEGFR, PDGFR, RAF1, KIT, and RET, leading to potent antitumor activity, such as the suppression of cell proliferation, apoptosis induction and the inhibition of angiogenesis [3]. Although this drug shows statistically significant prolongation of progression-free survival, its benefit does not last long, suggesting that tumor cells acquire chemoresistance to this compound. However, its molecular mechanism is mostly not yet clarified [3].

Recent progress in genetic perturbation technologies including the small interfering RNA (siRNA)/small hairpin RNA (shRNA) library and the clustered regularly interspaced short palindromic repeats (CRISPR) library, as an arrayed or pooled format, have enabled us to screen a large number of genes simultaneously. In particular, a pooled CRISPR/Cas9 library-based genetic screen is a versatile and convenient tool that can simultaneously assess the biological consequences of genetic loss-of-function for thousands of genes in a single experiment. Technically, the CRISPR library is introduced into the cell population under conditions of one gene perturbation per cell, and the entire cell population is exposed to selective pressure, which is determined based on the phenotype of interest. The cells that acquire adaptation to the applied environment upon gene knockout become enriched and are identified by high-throughput sequencing. While we and others have previously performed this screen for the discovery of cancer genes involving cell proliferation or tumor growth [4,5], it has also been applied to identify genes involved in drug susceptibility/resistance in several cancer types, including the BRAF inhibitor for melanoma and the FLT3 inhibitor for acute myeloid leukemia [6,7].

In this study, to discover genes involved in chemoresistance to regorafenib in HCC, we performed an in vivo pooled CRISPR library screen. Our screen successfully identified two paralogous kinases, LATS2 and LATS1, which are major components of the Hippo signaling pathway, as positive regulators of its antitumor effect. For the validation study, we showed that LATS2 inactivation dephosphorylated YAP, which in turn activated antiapoptosis and multidrug transporter machineries, mitigating the antitumor effect of regorafenib against HCC. Consistent with these results, we found that regorafenib efficacy was inversely correlated with the expression of YAP and downstream target genes in a variety of HCC cell lines. We also showed that the inhibition of YAP or its downstream antiapoptotic gene sensitized regorafenib-insensitive HCC cells to regorafenib. Our data suggest that the Hippo signaling pathway is the mediator of regorafenib efficacy in HCC.

## 2. Results

### 2.1. Regorafenib Suppresses Xenograft Tumor Growth of the Cas9-Expressing HCC Cell Line

**Figure 1:**
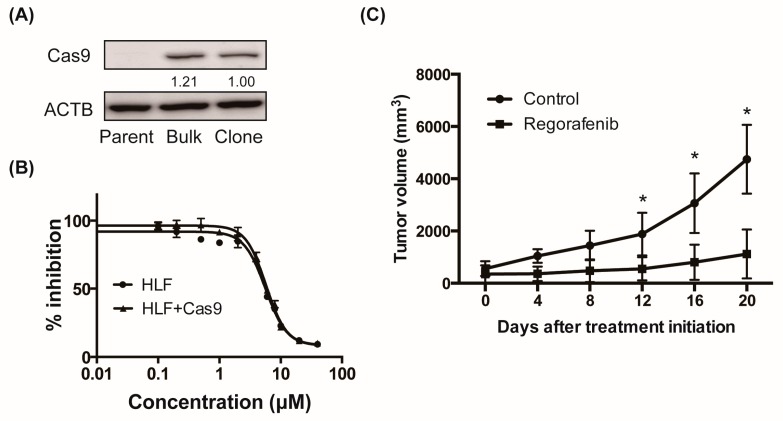
Regorafenib suppresses xenograft tumor growth of the Cas9-expressing hepatocellular carcinoma (HCC) cell line. (**A**) Western blot (WB) analysis of Cas9 protein levels in the HLF cell line. Parent stands for Cas9-nontransduced parental cells, Bulk stands for Cas9-transduced polyclonal cells, and Clone stands for Cas9-transduced monoclonal cells. Relative band intensity is shown. (**B**) Half maximal inhibitory concentration (IC50) values of regorafenib against HLF and HLF-Cas9 cells. (**C**) Nine million Cas9-transduced HLF clone cells per flank were subcutaneously injected into NOD/Shi-scid/IL-2Rγ^null^ (NOG) mice. Once the tumor volume reached 200 mm^3^, mice were treated with either vehicle or 20 mg/kg regorafenib, and tumor volume was measured at various times (*N* = 4 for each, * *p* < 0.05).

To perform a CRISPR screen, we first generated a Cas9-expressing human HCC cell line by lentiviral transduction of a Cas9-expressing vector into HLF cells and subsequent isolation of the Cas9-positive clone by the limiting dilution method. We confirmed that propagated clone cells expressed Cas9 protein at a similar level as those from transduced polyclonal cells (Figure 1A). We assessed the in vitro cytotoxic effect of regorafenib on these cells and confirmed that the half maximal inhibitory concentration (IC50) values of regorafenib were not affected by the presence of Cas9 (Figure 1B). Next, we examined the in vivo tumorigenic potential of the Cas9-positive HLF cell line and observed measurable xenograft tumors in the flanks of NOD/Shi-scid/IL-2Rγ^null^ (NOG) mice two months after inoculation. We then treated these mice with regorafenib or vehicle and confirmed that regorafenib significantly suppressed the growth of these tumors (Figure 1C). These data suggested that our in vivo experimental setting using the HCC xenograft model of Cas9-positive HLF clone cells, at least in part, mimicked the clinical antitumor effect of regorafenib on HCC patients. Therefore, we performed pooled CRISPR library screening using this model and aimed to discover genes involved in chemoresistance to regorafenib in HCC.

### 2.2. In Vivo Crispr Library Screen Identifies Lats1/2 Genes as Candidates Involved in Regorafenib Resistance in HCC

**Figure 2:**
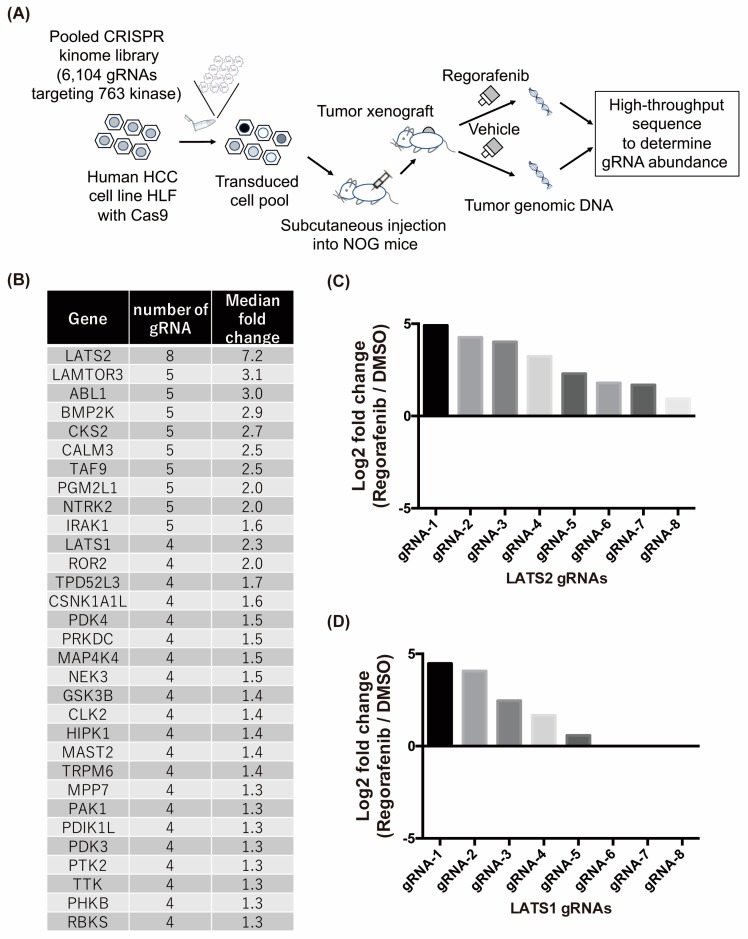
In vivo clustered regularly interspaced short palindromic repeats (CRISPR) library screen identifies LATS1/2 genes as candidates involved in regorafenib resistance in HCC. (**A**) Diagram depicting the in vivo pooled CRISPR library screen. (**B**) Thirty-one genes that positively affected regorafenib efficacy were identified by CRISPR screening. In the CRISPR kinome library, 8 unique gRNAs per gene were designed for 763 human kinases. The ratio of each gRNA abundance in regorafenib-treated tumors compared to that in vehicle-treated tumors was calculated, and genes with greater than or equal to 4 corresponding gRNAs showing 1.5-fold or more enrichment in regorafenib-treated tumors were selected. The number of gRNAs enriched in regorafenib-treated tumors and the median fold change of all 8 gRNAs are listed. (**C**,**D**) Log2 fold change values of each gRNA targeting LATS2 (**C**) or LATS1 (**D**) in regorafenib-treated tumors compared to those in vehicle-treated tumors are shown. Note that the fold change of gRNA6–8 in LATS1 was not calculated because of the absence of these gRNAs in vehicle-treated tumors.

For the CRISPR screen, we focused on the kinome. Lentiviral particles of the CRISPR pooed library containing 6104 gRNAs targeting 763 human kinases [8] were transduced into 2 × 10^7^ Cas9-positive HLF cells. After puromycin selection, in order to determine the initial distribution of gRNA abundance in the library-transduced cells, 9.0 × 10^6^ cells were collected and gRNA sequences were PCR amplified and sequenced by next-generation sequencing. A total of 6084 out of 6104 gRNAs were detected in transduced cancer cells with uniform distribution of their abundance, indicating the successful preparation of library-transduced cells (Supplementary Figure S1A). Then, 9.0 × 10^6^ transduced cancer cells per flank were subcutaneously inoculated into 6 NOG mice (Figure 2A). Once the tumor volume reached 200 mm^3^, tumors were randomly divided into two groups and treated with either vehicle or regorafenib for three weeks (Supplementary Figure S1B). After the treatment, all the tumors were collected and gRNA sequences integrated into the tumor genome were sequenced. An average of approximately 1.6 × 10^6^ gRNA sequences were obtained per tumor sample. Different from the distribution of gRNA abundance in library-transduced cells, the distribution in each tumor was uneven and some of gRNAs were highly enriched, suggesting the existence of strong in vivo selective pressure (Supplementary Figure S1A). We then expected that inside regorafenib-treated tumors, cancer cells that acquired resistance to regorafenib by CRISPR-mediated gene knockout would have a growth advantage and become enriched, while these cells would not become enriched inside vehicle-treated tumors. To identify genes targeted by CRISPR in those enriched cancer cells, we calculated the ratio of each gRNA abundance in regorafenib-treated tumors compared to that in vehicle-treated tumors and extracted 1048 gRNAs that showed greater than or equal to a 1.5-fold increase in ratio. We then examined genes with greater than or equal to 4 corresponding gRNAs identified among them and selected 31 genes, the knockout of which was expected to confer a growth advantage in cancer cells under regorafenib treatment (Figure 2B). Among them, LATS2, one of the Hippo signaling pathway components, drew our attention most because not only it showed biggest increase of gRNA abundance in regorafenib-treated tumors compared to that in vehicle-treated tumors (Figure 2B), but also the abundance of all designed 8 gRNAs was increased in regorafenib-treated tumors (Figure 2C). These data strongly suggest that HCC cells with the CRISPR-mediated knockout of LATS2 acquire regorafenib resistance. In addition, we also identified that the abundance of gRNAs targeting LATS1, another Hippo signaling pathway components and the paralogue of LATS2, was increased in regorafenib-treated tumors (Figure 2B,C), suggesting that the Hippo signaling pathway may be involved in the regorafenib resistance.

### 2.3. Inhibition of LATS2 Confers Regorafenib Resistance to HCC Cell Lines

**Figure 3:**
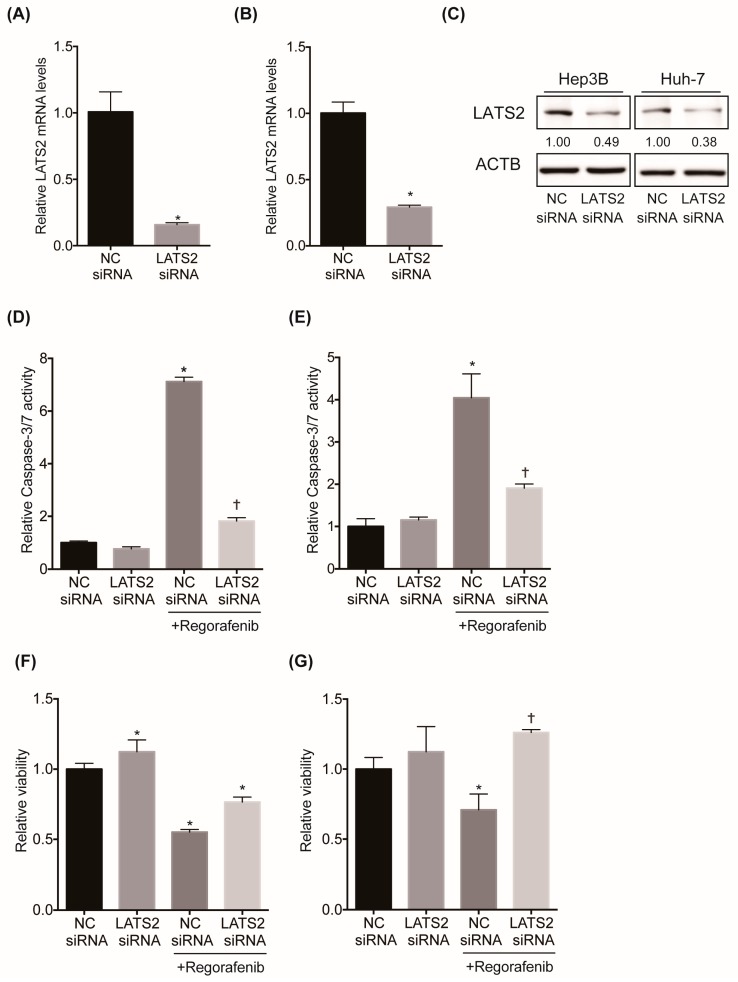
Inhibition of LATS2 confers regorafenib resistance to HCC cell lines. (**A**,**B**) LATS2 mRNA levels in Hep3B (**A**) and Huh-7 (**B**) cells 4 days after transfection with the negative control (NC) or LATS2 siRNA (*N* = 4 for each and * *p* < 0.05). (**C**) LATS2 protein levels in Hep3B and Huh-7 cells 4 days after transfection with the NC or LATS2 small interfering RNA (siRNA). Relative band intensity is shown (**D**,**E**). Three days after transfection with the NC or LATS2 siRNA, cells were treated with either DMSO or 2 μM regorafenib for 48 hours. Apoptosis was assessed by the caspase-3/7 activity of the culture supernatant in Hep3B (**D**) and Huh-7 (**E**) cells (*N* = 4 for each and *, † *p* < 0.05 vs. all). (**F**,**G**) Three days after transfection with the NC or LATS2 siRNA, cells were treated with either DMSO or 2 μM regorafenib for two days. Cell viability was measured by WST-1 assays in Hep3B (**F**) and Huh-7 (**G**) cells (*N* = 4 for each and * *p* < 0.05 vs. all and † *p* < 0.05 vs. NC siRNA and NC siRNA + regorafenib).

To validate the gene candidates identified in the CRISPR screen, we investigated the effect of LATS2 silencing on regorafenib-induced cytotoxicity and apoptosis in HCC cell lines in vitro. We first confirmed that the LATS2 siRNA significantly suppressed the mRNA and protein levels of LATS2 in 2 HCC cell lines, Hep3B and Huh7 (Figure 3A,C). While regorafenib treatment induced apoptosis, as exhibited by the marked increase in caspase-3/7 activity, it was significantly suppressed by LATS2 knockdown in both cell lines (Figure 3D,E). Similarly, while regorafenib treatment significantly impaired cell viability, as examined by the WST-1 assay, it was significantly alleviated by LATS2 knockdown in these cells (Figure 3F,G). These data indicate that the inhibition of LATS2 confers resistance to regorafenib in HCC cell lines.

### 2.4. Inhibition of LATS2 Inactivates the Hippo Signaling Pathway, Leading to the YAP-Mediated Activation of Downstream Machineries

**Figure 4:**
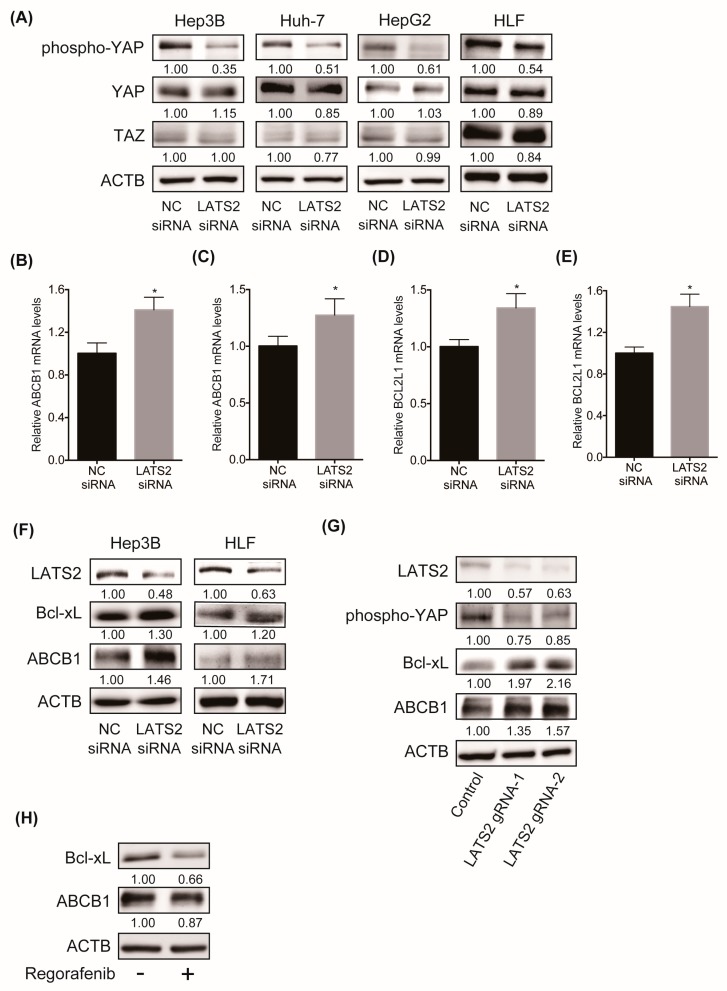
(**A**) WB analysis of phosphorylated YAP, whole YAP and TAZ protein levels in four human liver cancer cell lines four days after transfection with the NC or LATS2 siRNA. Relative band intensity is shown. (**B**,**C**) ABCB1 mRNA levels in Hep3B (**B**) and HLF (**C**) cells three days after transfection with the NC or LATS2 siRNA (*N* = 4 for each and * *p* < 0.05). (**D**,**E**) BCL2L1 mRNA levels in Hep3B (**D**) and HLF (**E**) cells three days after transfection with the NC or LATS2 siRNA (*N* = 4 for each and * *p* < 0.05). (**F**) WB analysis of the protein levels of LATS2, Bcl-xL, and ABCB1 in Hep3B and HLF cells five days after transfection with the NC or LATS2 siRNA. Relative band intensity is shown. (**G**) WB analysis of the protein levels of LATS2, phosphorylated YAP, Bcl-xL and ABCB1 in Cas9-expressing HLF cells transduced with negative control gRNA or LATS2 gRNA. Relative band intensity is shown. (**H**) WB analysis of the protein levels of Bcl-xL and ABCB1 in Hep3B cells treated with either DMSO or 2 μM regorafenib for 48 hours. Relative band intensity is shown.

We then examined the molecular mechanism of regorafenib resistance in HCC upon LATS2 inhibition. LATS2 and LATS1 are core components and positive regulators of Hippo signaling. When Hippo signaling is active, LATS2 and LATS1 phosphorylate the Hippo signaling mediator YAP, leading to its cytoplasmic retention and subsequent degradation [9]. Indeed, the siRNA-mediated knockdown of LATS2 reduced the levels of phosphorylated YAP in 4 human liver cancer cell lines (Figure 4A), proving that LATS2 negatively regulates YAP activity in liver cancer cell lines. Once YAP is activated by dephosphorylation, the YAP protein translocates to the nucleus and forms a complex with the transcriptional factor TEAD, leading to the upregulated expression of a variety of target genes [10]. We found that LATS2 inhibition activated YAP and transcriptionally upregulated the downstream targets of YAP/TEAD, the antiapoptotic gene Bcl-xL and the multidrug resistance transporter ABCB1 in Hep3B and HLF cells (Figure 4B–E). We also showed the upregulation of these proteins by western blot (WB) analysis (Figure 4F). A similar finding was observed, when LATS2 gene was depleted by CRISPR in Cas9-positive HLF cells using two LATS2-targeting gRNAs enriched in our CRISPR library screen (Figure 4G). We also found that regorafenib treatment reduced the levels of Bcl-xL and ABCB1 (Figure 4H). These data suggest that LATS2 silencing inactivates the Hippo signaling pathway, represented by YAP stabilization, and subsequently activates antiapoptotic and drug transporter machineries, which may contribute to regorafenib resistance in HCC cell lines.

### 2.5. Hippo Signaling May Mediate the Susceptibility of HCC Cells to Regorafenib

**Figure 5:**
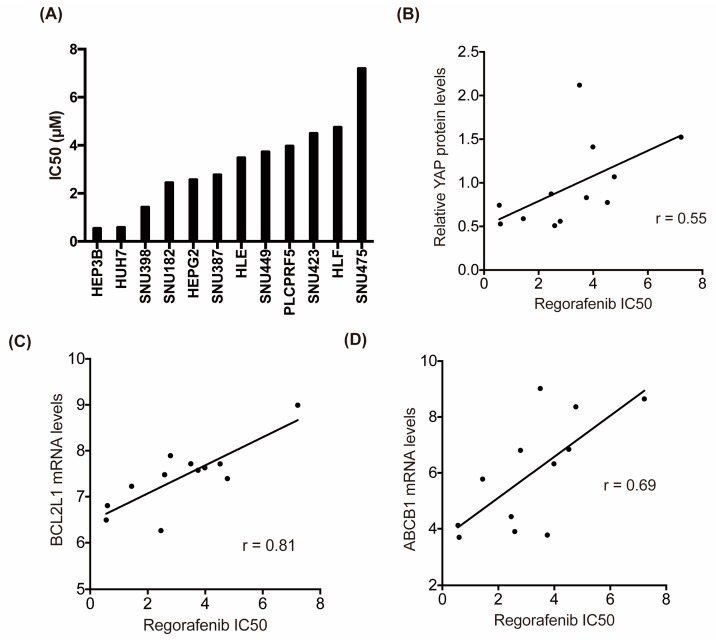
Hippo signaling activity may mediate the susceptibility of HCC cells to regorafenib. (**A**) IC50 values of regorafenib against 12 human liver cancer cell lines. (**B**–**D**) Correlation between the IC50 values of regorafenib and YAP protein levels (**B**) or BCL2L1 mRNA levels (**C**) or ABCB1 mRNA levels (**D**) against 12 human liver cancer cell lines. The Pearson correlation coefficient is shown as the r value.

To further examine the involvement of the Hippo signaling pathway in regorafenib efficacy in HCC, we investigated the association between YAP levels and regorafenib susceptibility in human liver cancer cell lines. We first determined the each IC50 values of regorafenib toward commercially available 12 human liver cancer cell lines (Figure 5A) and examined the correlation with their YAP protein levels. Interestingly, YAP protein levels showed a significant positive correlation with the IC50 values of regorafenib (Figure 5B). We also assessed the expression levels of CYR61, known downstream transcriptional target of YAP/TEAD component [11], as the marker of YAP activation. The mRNA levels of CYR61 show strong positive correlation with the IC50 values of regorafenib in 12 HCC cell lines (Supplementary Figure S2), suggesting that YAP activation may contribute to the regorafenib resistance. Furthermore, the mRNA levels of its downstream target genes, Bcl-xL and ABCB1, were also significantly positively correlated with the IC50 values of regorafenib in these HCC cell lines (Figure 5C,D). Collectively, these data suggest that Hippo signaling may mediate regorafenib susceptibility in HCC cells.

### 2.6. Suppression of YAP or Its Downstream Antiapoptotic Machinery Restores the Sensitivity of HCC Cells to Regorafenib

**Figure 6:**
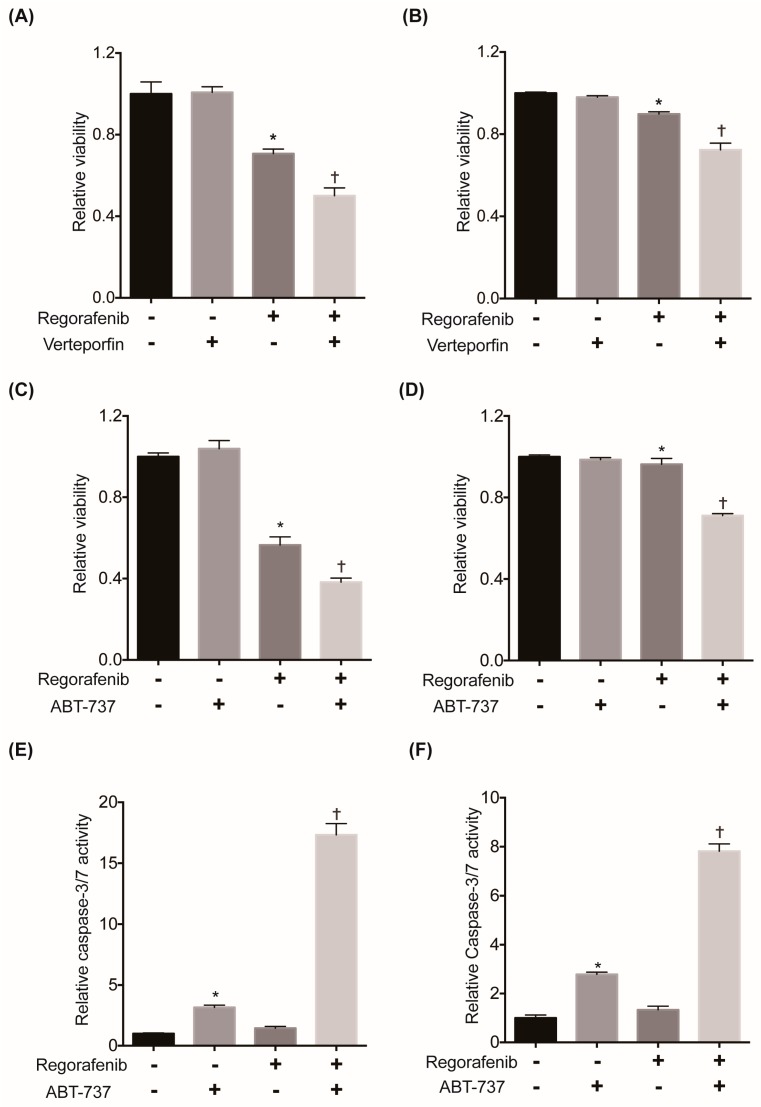
Suppression of YAP or its downstream antiapoptotic machinery restores regorafenib sensitivity in HCC cells. (**A**) Cells were treated with 15 μM regorafenib and/or 4 μM verteporfin for 48 hours. Cell viability was measured by WST-1 assays in HLF (*N* = 4 for each and *, † *p* < 0.05 vs. all). (**B**) Cells were treated with 10 μM regorafenib and/or 2 μM verteporfin for 48 hours in SNU-475 cells. Cell viability was measured by WST-1 assays (*N* = 4 for each and *, † *p* < 0.05 vs. all). (**C**) Cells were treated with 15 μM regorafenib and/or 4 μM ABT-737 for 48 hours. Cell viability was measured by WST-1 assays in HLF cells (*N* = 4 for each and *, † *p* < 0.05 vs. all). (**D**) Cells were treated with 7 μM regorafenib and/or 1 μM ABT-737 for 24 hours. Cell viability was measured by WST-1 assays in SNU-475 cells (*N* = 4 for each and * *p* < 0.05 vs. vehicle and regorafenib + ABT-737, † *p* < 0.05 vs. all). (**E**) Cells were treated with 15 μM regorafenib and/or 4 μM ABT-737 for 24 hours. Apoptosis was assessed by the caspase-3/7 activity of the culture supernatant in HLF cells (*N* = 4 for each and *, † *p* < 0.05 vs. all). (**F**) Cells were treated with 7 μM regorafenib and/or 1 μM ABT-737 for 24 hours. Apoptosis was assessed by the caspase-3/7 activity of the culture supernatant in SNU-475 cells (*N* = 4 for each and *, † *p* < 0.05 vs. all).

To clarify the causal relationship between YAP activation and regorafenib susceptibility, we examined the effect of suppressing YAP on the cytotoxicity of regorafenib in HCC cells. We selected HLF and SNU-475 cells, which showed the highest IC50 values of regorafenib and YAP among the 12 HCC cell lines, and treated them with the YAP inhibitor verteporfin. While verteporfin treatment alone did not affect cell viability, YAP inhibition significantly augmented the cytotoxicity of regorafenib in these cells (Figure 6A,B). We also confirmed that siRNA-mediated YAP knockdown also significantly augmented the cytotoxicity of regorafenib (Supplementary Figure S3A). These findings demonstrated that YAP activation contributed to chemoresistance to regorafenib in HCC cells. We also examined the involvement of the YAP downstream target gene Bcl-xL in regorafenib efficacy. Similar to YAP inhibition, Bcl-xL inhibition by ABT-737, a Bcl-xL/Bcl-2/Bcl-w inhibitor, significantly enhanced cytotoxicity induced by regorafenib (Figure 6C,D) in HLF and SNU-475 cells. We also observed a significant elevation in caspase-3/7 activity in both cell lines when treated with both regorafenib and ABT-737 compared to treatment with either single agent (Figure 6E,F). We also confirmed that siRNA-mediated Bcl-xL knockdown significantly enhanced the cytotoxicity of regorafenib with marked elevation of caspase-3/7 activity (Supplementary Figure S3B,C). Collectively, these data also suggest that the suppression of YAP or its downstream antiapoptotic machinery in combination with regorafenib could be a potential therapeutic for regorafenib-insensitive HCC cell lines.

## 3. Discussion

In the present study, through CRISPR-based high-throughput screening, we discovered that Hippo signaling was involved in chemoresistance to regorafenib in HCC. As a mechanism of action, we showed that inactivation of the Hippo signaling pathway activated YAP, upregulating machineries involved in antiapoptosis and drug resistance transporters. Last, we proposed the potential combination therapy of regorafenib and the inhibition of YAP or Bcl-xL for regorafenib-insensitive HCC patients.

Although regorafenib is also used for advanced colorectal cancer and gastrointestinal stromal tumors (GISTs) other than HCC, there are only a few reports regarding its chemoresistance mechanism. Mirone G. et al. reported that Notch-1 was upregulated in regorafenib-resistant colon cancer cells and that its inhibition partially restored regorafenib sensitivity [12]. Tong J. et al. reported that the inactivating mutation in FBW7, an E3 ubiquitin ligase, contributes to regorafenib resistance in colorectal cancer cells [13]. They also demonstrated that it is caused by stabilization of the antiapoptotic protein Mcl-1 due to the lack of FBW7-mediated proteasomal degradation. For HCC, Wang J. et al. recently reported that Pin1 expression was increased in a regorafenib-resistant HCC cell line and that its suppression reversed regorafenib resistance [14]. Mechanistically, they showed that Pin1 positively regulated EMT and the migration/invasion capacity of HCC cells. In the current paper, we newly identified that the Hippo signaling pathway is involved in regorafenib chemoresistance in HCC. In addition, similar to Tong’s study, we found that the activation of antiapoptotic Bcl-2 family proteins contributed to its chemoresistance mechanism.

The Hippo signaling pathway is the master regulator of organ development and also plays a critical role in liver size control [15,16]. In addition, a substantial number of papers has elucidated the important role of Hippo signaling in tumorigenesis in a variety of cancers, including HCC [15,16]. Genetic studies have shown that the liver-specific deletion of MST1/2, another core component of the Hippo signaling pathway, or YAP overexpression induces liver overgrowth and the development of HCC in mice [17,18]. We also previously demonstrated that the liver-specific deletion of Sav1, an adaptor protein of MST1/2, activated YAP and significantly accelerated liver tumor development in the Pten-deficient liver [9]. The Hippo signaling pathway is now appreciated as one of the major gatekeepers of hepatocarcinogenesis. Moreover, the association between Hippo signaling and resistance to chemotherapeutic drugs has been recently received great attention [19,20,21]. YAP is involved in resistance to a variety of chemotherapeutic drugs in several cancer types, including molecular targeted drugs such as inhibitors of CDK4/6, EGFR and BRAF [22,23,24], as well as classical cytotoxic drugs such as 5-FU, cisplatin (CDDP) and doxorubicin [25,26,27]. Regarding HCC, YAP contributes to chemoresistance to doxorubicin [28], CDDP [29], and irinotecan [30]. Gao J, et al. also recently reported that tissue stiffness observed in cirrhotic liver induced sorafenib resistance via the upregulation of YAP [31]. In regard to its mechanism of action, YAP contributed to chemoresistance through the induction of autophagy, cell cycle, antiapoptosis, and EMT [21]. In the current paper, we showed for the first time that the Hippo pathway and its effector YAP contributed to regorafenib resistance in HCC in vitro. We found that it was in part through the induction of antiapoptosis and multidrug transporter machineries. But other chemoresistance mechanisms could be also involved and need further investigation. In addition, considering that our screening was done in vivo and regorafenib affects VEGF/PDGF signaling, YAP activation might confer regorafenib resistance in HCC cells through affecting tumor vasculature and microenvironment. An in vivo study to validate our finding and pursue these interactions is desired and will be performed.

In addition to LATS1 and LATS2 proteins, MST1 and MST2 proteins, known as STK3 and STK4, are also core components of the Hippo signaling pathway. They are upstream kinases of LATS1/2 and thus physiologically suppress YAP activity same as LATS1/2 do. In our screen, although all four kinases were targeted by the CRISPR kinome library, we only identified the enrichment of gRNAs targeting LATS1/2 but not MST1/2 in regorafenib-treated tumors. The definitive reason of discordancy is unclear but there could be due to the distinct strength of these proteins regulating YAP activity. It was shown that in MST1/2 knockout hepatocytes, there is residual phosphorylation of YAP at LATS1/2 phosphorylation sites suggesting that LATS1/2 is still active and suppress YAP activity in the absence of MST1/2 [32]. Moreover, YAP induces a negative feedback loop through directly enhancing the transcription of LATS1/2 [33]. As LATS1/2 deletion would lose this negative feedback loop, higher levels of YAP activation in LATS1/2-deficient cells would be anticipated relative to depletion of other upstream Hippo pathway components such as MST1/2. Although there is no direct experimental evidence to support, such difference between LATS1/2 and MST1/2 molecules could affect the result of our CRISPR screen.

In addition to the limited survival benefit of regorafenib, its response rate is unsatisfactory. Therefore, it is urgent to identify efficacious biomarkers for the selection of good responders. Teufel M. et al. recently analyzed plasma and tumor samples from HCC patients who participated in the RESORCE trial [34]. Among 266 evaluable proteins in the plasma of 499 patients, the levels of 5 (ANG-1, cystatin-B, LAP TGF-β1, LOX-1, and MIP-1α) were negatively associated with an enhanced treatment benefit with regorafenib, with a hazard ratio between 1.02 and 1.46. They also showed that some of the circulating miRNA levels were also significantly correlated with overall survival. Although very important results were obtained from a large cohort of patients, their predictive value is still uncertain until proven by further validation studies. In our current study, we revealed that tumor Hippo signaling activity was closely associated with the regorafenib response. Therefore, it might be worth further investigation to identify surrogate serum/plasma biomarkers for Hippo signaling pathway activity in HCC tissue, which could potentially be used for the identification of efficacious biomarkers of regorafenib in HCC. Meanwhile, although we could show that LATS2 depletion functionally confers regorafenib resistance in the HCC cells, it does not necessarily mean that HCC cells acquire chemoresistance by inactivating LATS2 in the clinical setting. It could be achieved by activation of YAP by other mechanism. Therefore, future studies are also important to assess the alteration of these signals in HCC specimens from patients who become refractory to regorafenib and/or regorafenib-resistant HCC cell lines.

In conclusion, in the current study, we discovered an unprecedented link between the Hippo signaling pathway and regorafenib efficacy in HCC. Further studies are required to evaluate the potential of Hippo signaling as a candidate biomarker for efficacy against regorafenib in HCC patients and as a therapeutic target for patients with regorafenib-insensitive HCC.

## 4. Materials and Methods

All methods were performed in accordance with the relevant guidelines and regulations of our institution.

### 4.1. Cell Lines and Reagents

Human liver cancer cell lines Hep3B, PLC/PRF/5, HepG2, SNU-182, SNU-387, SNU-398, SNU-423, SNU-449, and SNU-475, HLE and HLF were purchased from the American Type Culture Collection (ATCC, Manassas, VA, USA). The Huh-7 human liver cancer cell line was obtained from the Japanese Cancer Research Resource Bank (JCRB, Ibaraki, Japan). These cell lines were cultured in RPMI-1640 medium or Dulbecco’s modified eagle medium (DMEM) supplemented with 10% fetal calf serum and antibiotics. All cells were confirmed to be free from pathogens and mycoplasma. Regorafenib was provided by Bayer AG (Leverkusen, Germany). Regorafenib was dissolved in the mixture of PEG400, propylene glycol and pluronic F68. ABT-737 and Verteporfin were purchased from Cayman Chemical (Ann Arbor, MI, USA). These compounds were dissolved in dimethyl sulfoxide (DMSO) to prepare stock solutions and diluted in cell growth medium for assays.

### 4.2. Production of Lentiviral Particles Containing the Human Kinome CRISPR Knockout Library (Brunello) or Lentiviral Vectors

We obtained the Brunello library from Addgene. The two halves of the library were combined and used as one pool containing 6104 gRNAs targeting 763 genes (approximately 8 gRNAs per gene), it was electroporated and transformed following the protocol from the Broad institute [8]. To produce lentivirus, 180 µg of the lentiCRISPR plasmid library, 90 µg of pVSVg (Addgene, Watertown, MA, USA) and 126 µg of psPAX2 (Addgene) in OptiMEM (Thermo Fisher Scientific, Waltham, MA, USA) were mixed with 900 µL of Lipofectamine 2000 (Thermo Fisher Scientific). Then, the mixture was added to 2.5 × 10^8^ HEK293 cells. Sixty-four hours later, the supernatant was collected and concentrated using a Lenti-X Concentrator (Takara Bio, Kusatsu, Japan) and stored at −80 °C.

The production of lentiviral particles containing a single vector was performed as follows: For the Cas9-expressing vector, the lentiCas9-Blast vector was obtained from Addgene. To produce lentivirus, 5 µg of lentiGuide-Puro or lentiCas9-Blast, 2.5 µg of pVSVg and 3.5 µg of psPAX2 in OptiMEM were mixed with 25 µL of Lipofectamine 2000. Then, the mixture was added to 6.5 × 10^6^ HEK293 cells. Forty-eight hours later, the supernatant was collected and concentrated using a Lenti-X Concentrator and stored at −80 °C.

### 4.3. Generation of Cas9-Positive HLF Monoclonal Cells and LATS2-Depleted Cells by CRISPR

HLF cells were plated at a density of 1.5 × 10^5^ cells per 6-well plate one day before infection. The next day, the medium was replaced with serum-free medium containing 4 µg/mL polybrene (Merck Millipore, Burlington, MA, USA) and transduced with lentivirus containing lentiCas9-Blast at a multiplicity of infection (MOI) of 10. The following day, cells were supplemented with complete medium containing 4.0 μg/mL blasticidin (Fujifilm Wako pure chemical corporation, Osaka, Japan) for 7 days. Then, several single clones were established by the limiting dilution method, and Cas9 expression in these cells was confirmed by WB analysis and flow cytometry.

To generate LATS2-depleted cells by CRISPR, Cas9-positive HLF cells were plated at a density of 1.5 × 105 cells per 6-well plate one day before infection. The next day, the medium was changed into serum-free medium containing 8 µg/mL of polybrene and transduced with lentivirus containing lentiguide-puro vector cloned with gRNA targeting LATS2 (gRNA-1: ACCAGCAGAAGGTTAACCGG, gRNA-2: ATAAGGTCCGAACTTTGGGG) at an MOI of 2–3. The following day, cells were supplemented with complete medium containing 1.0 μg/mL puromycin (Thermo Fisher Scientific) for 7 days.

### 4.4. In Vivo Pooled CRISPR Kinome Library Screening

Based on library size, all the following steps including infection, puromycin selection, injection and gDNA isolation were performed with enough cells to maintain a representation of more than 200 cells per gRNA in the library. Twenty million HLF cells expressing Cas9 were transduced with the Brunello library [8] at an MOI of 0.2. After 7 days of the selection of transduced cells by puromycin, 9.0 × 10^6^ library-transduced cells were injected into the flanks of NOG mice and also stocked for subsequent gDNA isolation. Mice were monitored for tumor growth at least twice a week. Tumor volume was calculated by the following formula: volume (mm^3^) = length × width × width × 0.52. Once each tumor volume reached 200 mm^3^, tumors were randomly divided into two groups and treated with either vehicle or 20 mg/kg regorafenib for three weeks. Then, all the tumors (three tumors from the vehicle group and three tumors from the regorafenib group) were collected. All xenograft procedures were approved by the Institutional Animal Care and Use Committee (IACUC) at Osaka University Graduate School of Medicine (30-015-023).

Genomic DNA from transduced cells and xenografted tumors was extracted using a DNeasy Blood and Tissue Kit (Qiagen, Germantown, MD, USA) with RNase treatment. gRNA sequences integrated into the gDNA were amplified using illumina-adapted forward and reverse indexed PCR primers according to the protocol from the Broad institute [8] and purified with a PCR purification kit (Qiagen). A total of 8.0 μg of gDNA per sample was used for the PCR. Purified amplicons were multiplexed and sequenced in Illumina HiSeq. We obtained an average of 1.6 × 10^6^ assigned gRNA sequences per cells or tumor, indicating that the library complexity was maintained more than 200× throughout the entire experiment. Log2 normalized read counts of each gRNA were plotted to determine the distribution of gRNA abundance inside cells and tumors (Supplementary Figure S1A). We then calculated the ratio of each gRNA abundance in regorafenib-treated tumors compared to that in vehicle-treated tumors (Supplementary Table S1).

### 4.5. siRNA Transfection

The siRNA oligonucleotides were purchased from Thermo Fisher Scientific. The silencer select siRNA IDs are as follows; negative control No.1 siRNA (Catalog number 4390843) and LATS2(s25504). The siRNA was transfected into human liver cancer cell lines using Lipofectamine RNAiMAX reagent (Thermo Fisher Scientific) according to the manufacturer’s protocol. Two to three days after transfection, knockdown efficiency was assessed by qRT-PCR.

### 4.6. Cell Viability Assay

Cell viability was assessed using WST-1 reagent (Takara Bio) according to the manufacturer’s protocol. Briefly, 3 × 10^3^ to 8 × 10^3^ cells were seeded into 96-well plates with or without siRNA transfection and cultured with medium containing 2–10% fetal calf serum with or without the compounds of interest for 1–5 days. WST-1 reagent was added to each well and incubated for 1.5 h at 37 °C. The absorbance at 450 nm and 650 nm (reference) was measured using a micro plate reader SH-9000Lab (CORONA).

### 4.7. Caspase-3/7 Activity

Caspase-3/7 activity of the cell supernatant was measured with a luminescence substrate assay for caspase-3 and caspase-7 (Caspase-Glo assay, Promega) as described previously [35].

### 4.8. Real-Time PCR

Total RNA was extracted from cells or tumor tissues using a RNeasy Plus Mini Kit (Qiagen) according to the manufacturer’s instructions. RNA was reverse transcribed using ReverTra Ace qPCR Master Mix (TOYOBO, Osaka, Japan). qPCR was performed using the QuantStudio 6 Flex RT-PCR system with TaqMan Gene Expression Assay probes (Thermo Fisher Scientific). The TaqMan probe IDs are as follows; ABCB1 (Hs00184500_m1), ACTB (Hs01060665_g1), BCL2L1 (Hs00236329_m1), and LATS2 (Hs01059009_m1).

### 4.9. Western Blot Analysis

The cell pellet or xenograft tissue was lysed in lysis buffer (1% Nonidet P-40, 0.5% sodium deoxycholate, 0.1% sodium dodecyl sulfate, 1 × protease inhibitor cocktail (Nacalai Tesque, Kyoto, Japan), 1 × phosphatase inhibitor cocktail (Nacalai Tesque) and phosphate-buffered saline, pH 7.4). Protein concentrations were measured using a bicinchoninic acid protein assay kit (Pierce). Equal amounts of protein lysates were electrophoretically separated using sodium dodecyl sulfate (SDS) polyacrylamide gels and transferred onto a polyvinylidene fluoride membrane. After incubation in 5% dry milk in Tris-buffered saline (TBS) and 0.1% Tween 20 for 1 h at room temperature (RT), the membranes were incubated with the primary antibodies overnight at 4 °C. The following primary antibodies were used: Rabbit polyclonal antibody to Bcl-xL (Santa Cruz Biotechnology), rabbit polyclonal antibody to phospho-YAP (Cell Signaling Technology), rabbit polyclonal antibody to YAP (Cell Signaling Technology), rabbit polyclonal antibody to TAZ (Cell Signaling Technology), rabbit polyclonal antibody to ABCB1 (Cell Signaling Technology), rabbit polyclonal antibody to Cas9 (Cell Signaling Technology), rabbit polyclonal antibody to LATS2 (Abcam), and rabbit polyclonal antibody to ACTB (Sigma-Aldrich, St. Louis, MO, USA). After incubation with the secondary antibody for 1 hour, immunolabelled proteins were detected using a chemiluminescent substrate (Pierce). Intensity of each band is measured by densitometry and normalized by intensity of corresponding β-actin band using Image-J software (version 1.52). Detailed information can be found at Figure S4.

### 4.10. Correlation Between the IC50 Values of Regorafenib and mRNA Levels in Human Liver Cancer Cell Lines

The IC50 values of regorafenib in 12 human liver cancer cell lines were calculated based on the WST-1 cell viability assay. Microarray data of cancer cell lines using Affymetrix U133 + 2 arrays obtained in the Cancer Cell Line Encyclopedia (CCLE) project [36] are publicly available and were downloaded from the CCLE (https://portals.broadinstitute.org/ccle). The Pearson correlation coefficient between the IC50 value and each mRNA level was calculated and is presented as the r value.

### 4.11. Statistics

All data are presented as the mean ± standard deviation (S.D.). Statistical analyses were performed using an unpaired Student’s *t* test or one-way analysis of variance (ANOVA) in GraphPad Prism^®^ 6 software (version 6.0f). When ANOVA was applied, the differences in the mean values among the groups were examined by post hoc correction. The Pearson correlation coefficient was calculated in GraphPad Prism^®^ 6, and *p* < 0.05 was considered statistically significant.

## 5. Conclusions

CRISPR loss-of-function screen identified the Hippo signaling pathway as the mediator of regorafenib efficacy in HCC.

## Supplementary Materials

The following are available online at https://www.mdpi.com/2072-6694/11/9/1362/s1. Figure S1: The distribution of gRNA abundance and tumor growth curve during CRISPR library screening, Figure S2: Correlation between YAP activation and regorafenib resistance, Figure S3: Genetic suppression of YAP or Bcl-xL restores regorafenib sensitivity in HCC cells, Figure S4: Original images of western blot with molecular weight markers, Table S1: The ratio of each gRNA abundance in regorafenib-treated tumors compared to that in vehicle-treated tumors

## Abbreviations

ATCC	American Type Culture Collection
CCLE	Cancer Cell Line Encyclopedia
CDDP	cisplatin
gRNA	guide RNA
CRISPR	clustered regularly interspaced short palindromic repeats
DMEM	Dulbecco’s modified eagle medium
FDA	Food and Drug Administration
GISTs	gastrointestinal stromal tumors
HCC	hepatocellular carcinoma
IACUC	the Institutional Animal Care and Use Committee
IC50	half maximal inhibitory concentration
JCRB	Japanese Cancer Research Resource Bank
MOI	multiplicity of infection
NOG	NOD/Shi-scid/IL-2Rγ^null^
siRNA	small interfering RNA
shRNA	small hairpin RNA
WB	western blot
