# Supplementary material for: CRISPR Loss-of-Function Screen Identifies the Hippo Signaling Pathway as the Mediator of Regorafenib Efficacy in Hepatocellular Carcinoma
Source: Cancers (Basel). 2019 Sep 13;11(9):1362. doi: 10.3390/cancers11091362 (PMC6770429; doi:10.3390/cancers11091362)
Supplement: Supplementary file 1 [file cancers-11-01362-s001.zip › cancers-581270-supplementary/cancers-581270 - supplementary - final check/cancers-581270-supplementary_proof.pdf]

cancers

MDPI

Article

CRISPR Loss-of-Function Screen Identifies the Hippo Signalling Pathway as the Mediator of Regorafenib Efficacy in Hepatocellular Carcinoma

Shigeki Suemura , Takahiro Kodama , Yuta Myojin, Ryoko Yamada, Minoru Shigekawa, Hayato Hikita, Ryotaro Sakamori, Tomohide Tatsumi and Tetsuo Takehara

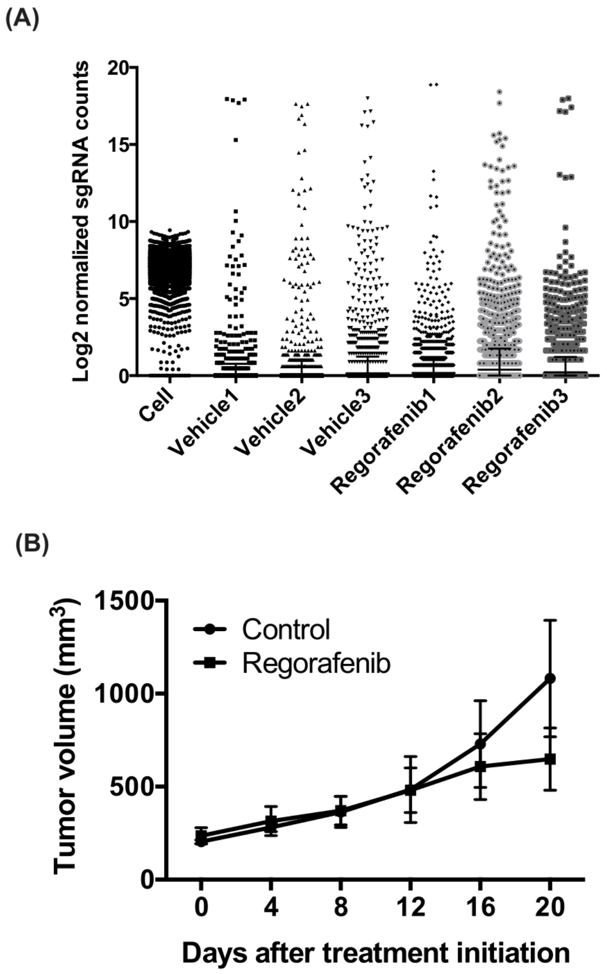

Figure S1. The distribution of gRNA abundance and tumour growth curve during CRISPR library screening. Twenty million HLF cells expressing Cas9 were transduced with the Brunello library at an MOI of 0.2. After 7 days of the selection of transduced cells by puromycin, 9.0 × 106 library-transduced cells were injected into the flanks of NOG mice and also stocked for subsequent gDNA isolation. Once each tumour volume reached 200 mm3, tumours were randomly divided into 2 groups and treated with either vehicle or 20 mg/kg regorafenib for 3 weeks. Then, all the tumours (3 tumours from the vehicle group and 3 tumours from the regorafenib group) were collected. Genomic DNA from transduced cells and xenografted tumours was extracted. gRNA sequences integrated into the gDNA were PCR amplified and sequenced in Illumina HiSeq. (**A**) Log2 normalized read counts of each gRNA were plotted to determine the distribution of gRNA abundance inside cells and tumours (**B**) Tumour volumes were measured every 4 days after treatment initiation.

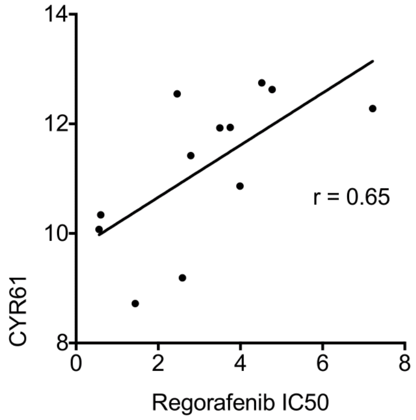

Figure S2. Correlation between YAP activation and regorafenib resistance. Correlation between the IC50 values of regorafenib and CYR61 mRNA levels against 12 human liver cancer cell lines. The Pearson correlation coefficient is shown as the r value.

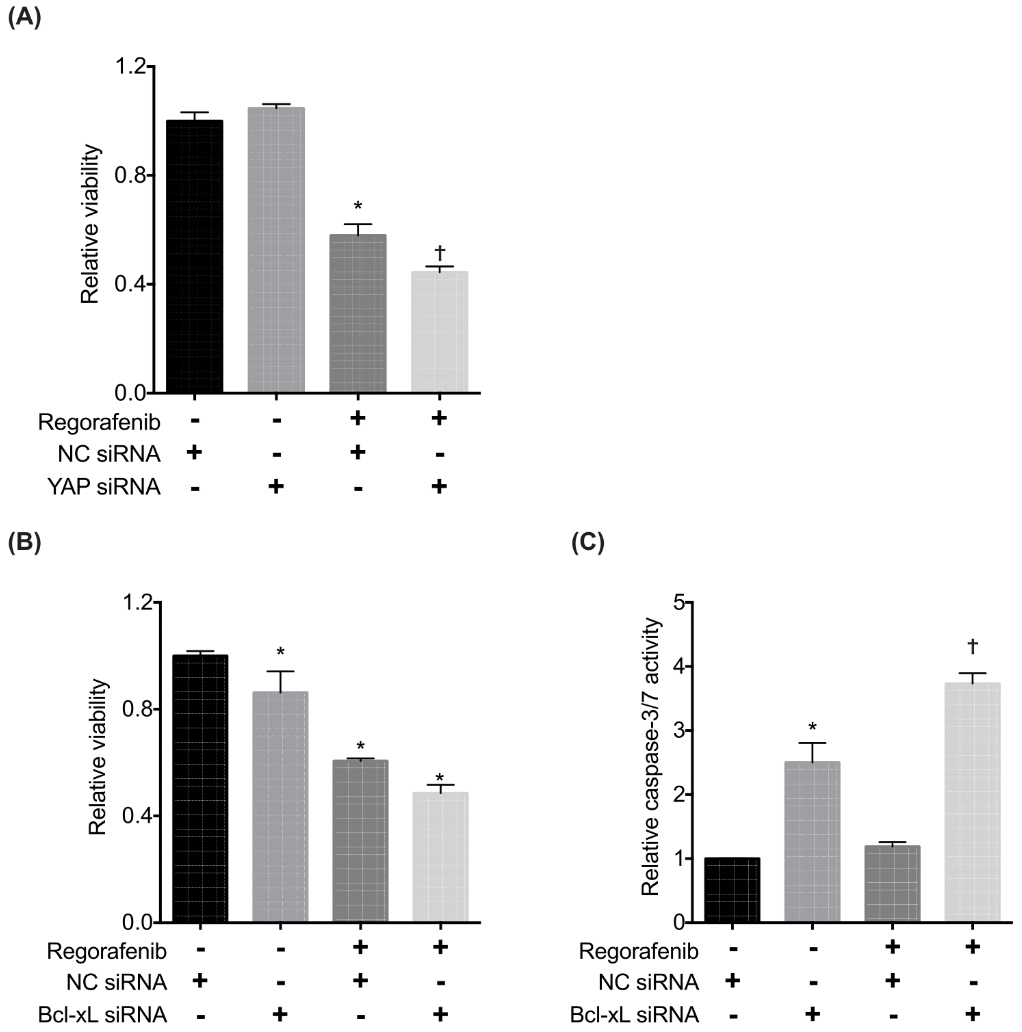

Figure S3. Genetic suppression of YAP or Bcl-xL restores regorafenib sensitivity in HCC cells. (**A**) Three days after transfection with the NC or YAP siRNA, cells were treated with DMSO or 15 μM regorafenib for 48 hours. Cell viability was measured by WST-1 assays in HLF (N = 4 for each and *, † $p < 0.05$ vs all)**.** (**B,C**) Three days after transfection with the NC or Bcl-xL siRNA, cells were treated with DMSO or 15 μM regorafenib for 48 hours. Cell viability was measured by WST-1 assays (**B**) and apoptosis was assessed by the caspase-3/7 activity of the culture supernatant (**C**) in HLF (N = 4 for each and *, † $p < 0.05$ vs all)**.**

Figure 1A

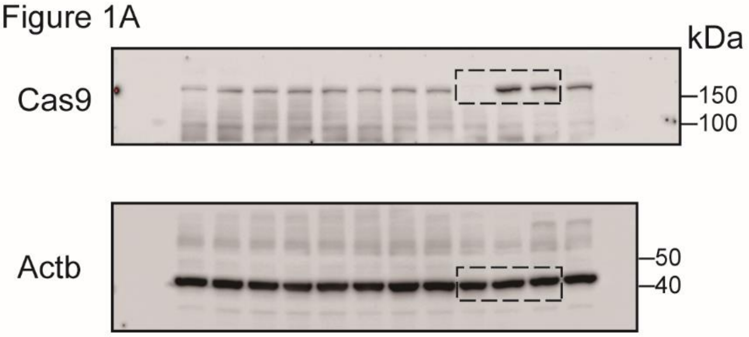

Figure 3C

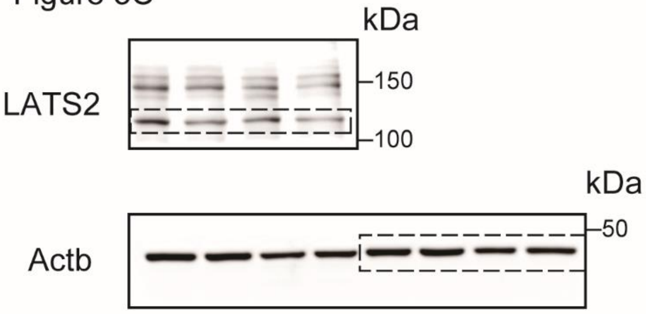

Figure 4A HLF

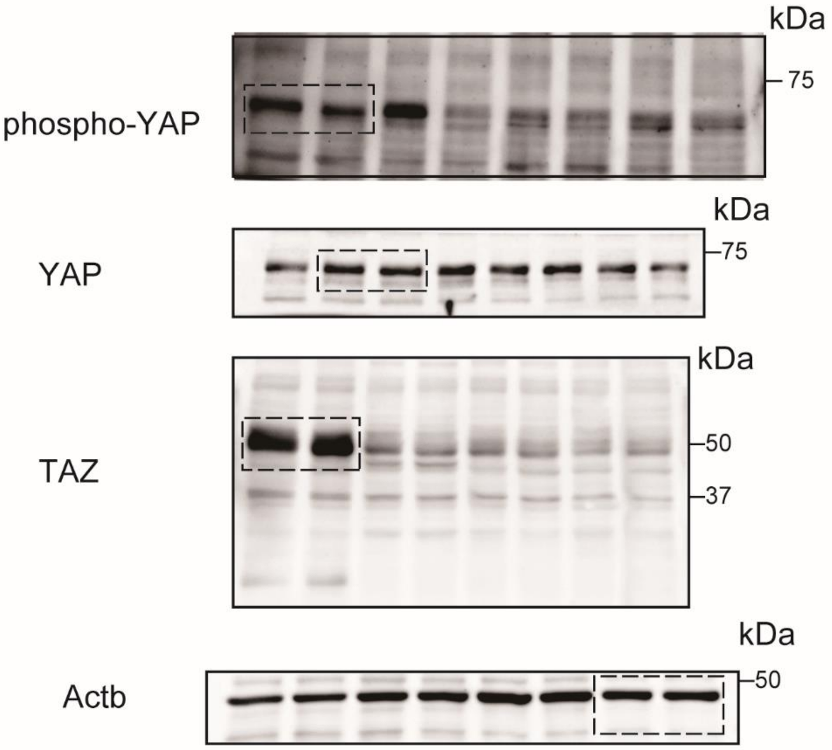

Figure 4A Hep3B

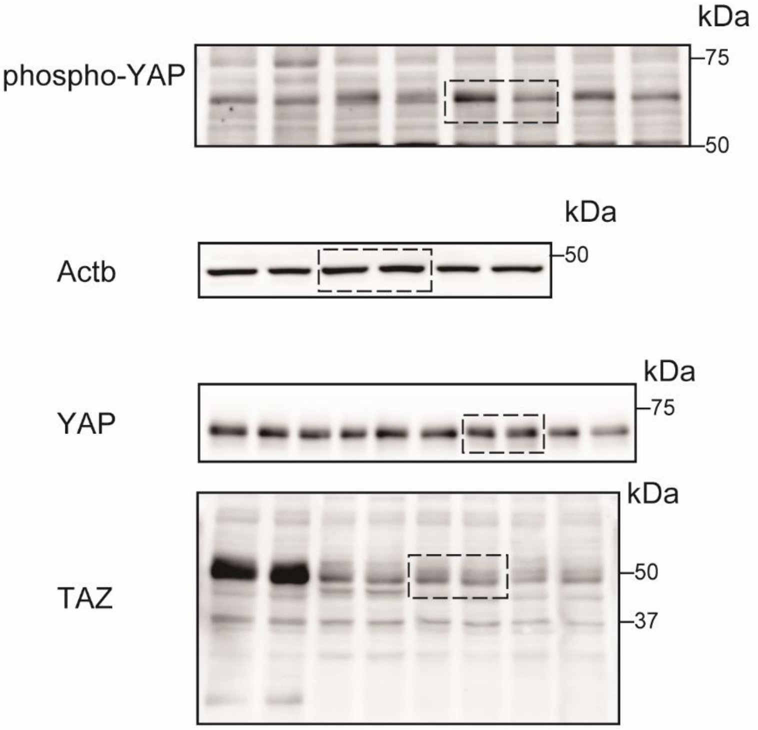

Figure 4A Huh-7

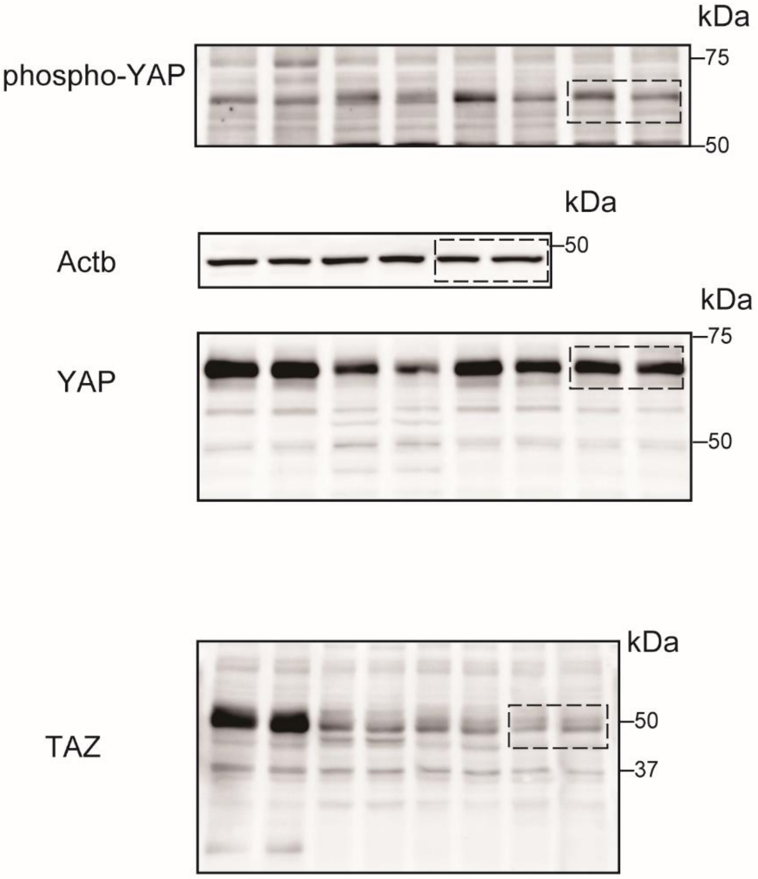

Figure 4A

HepG2

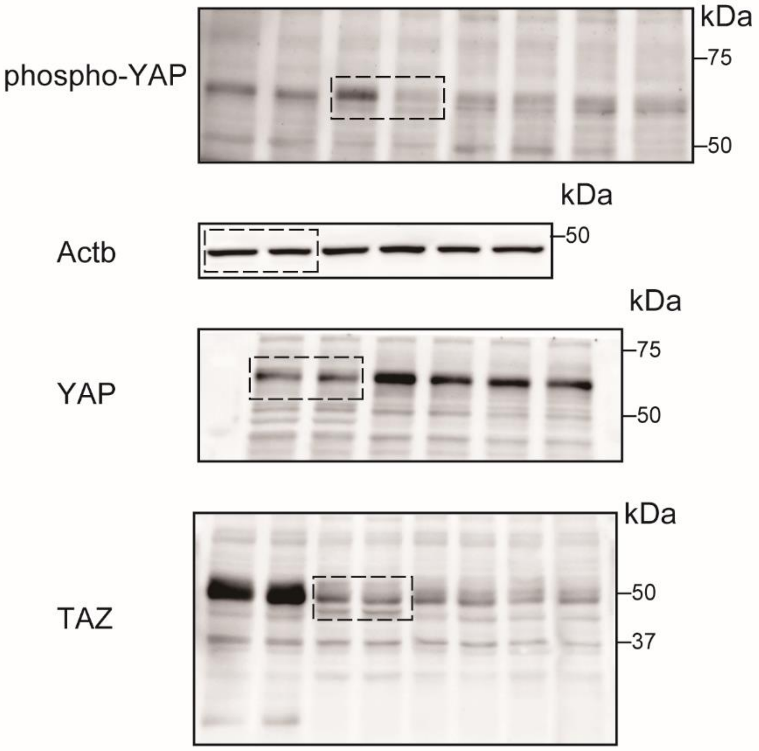

Figure 4F

Hep3B

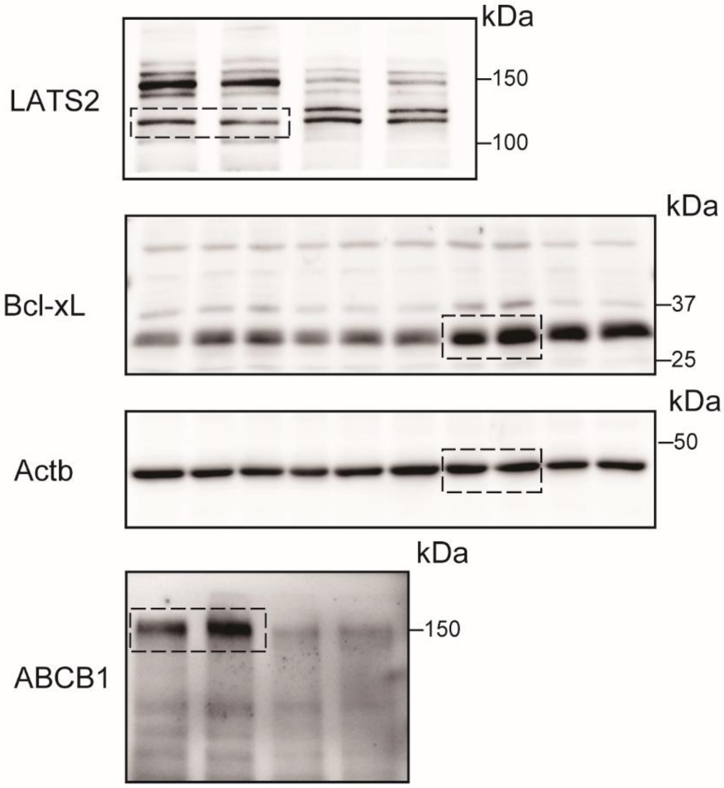

Figure 4F

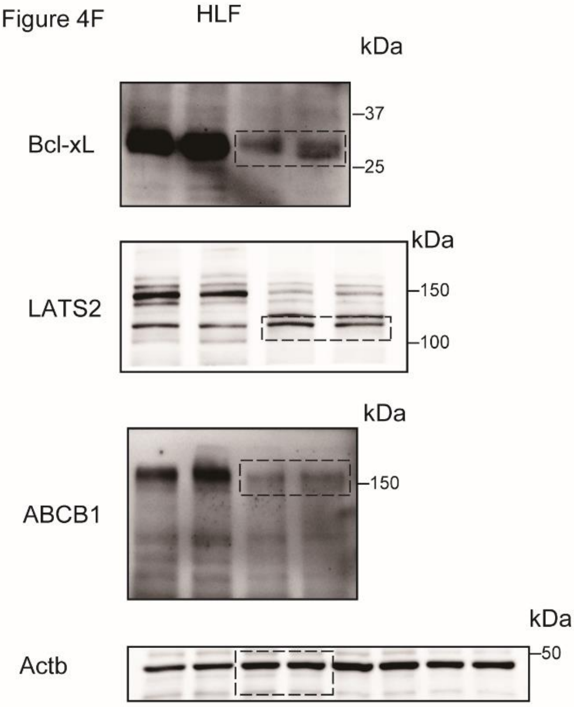

Figure 4G

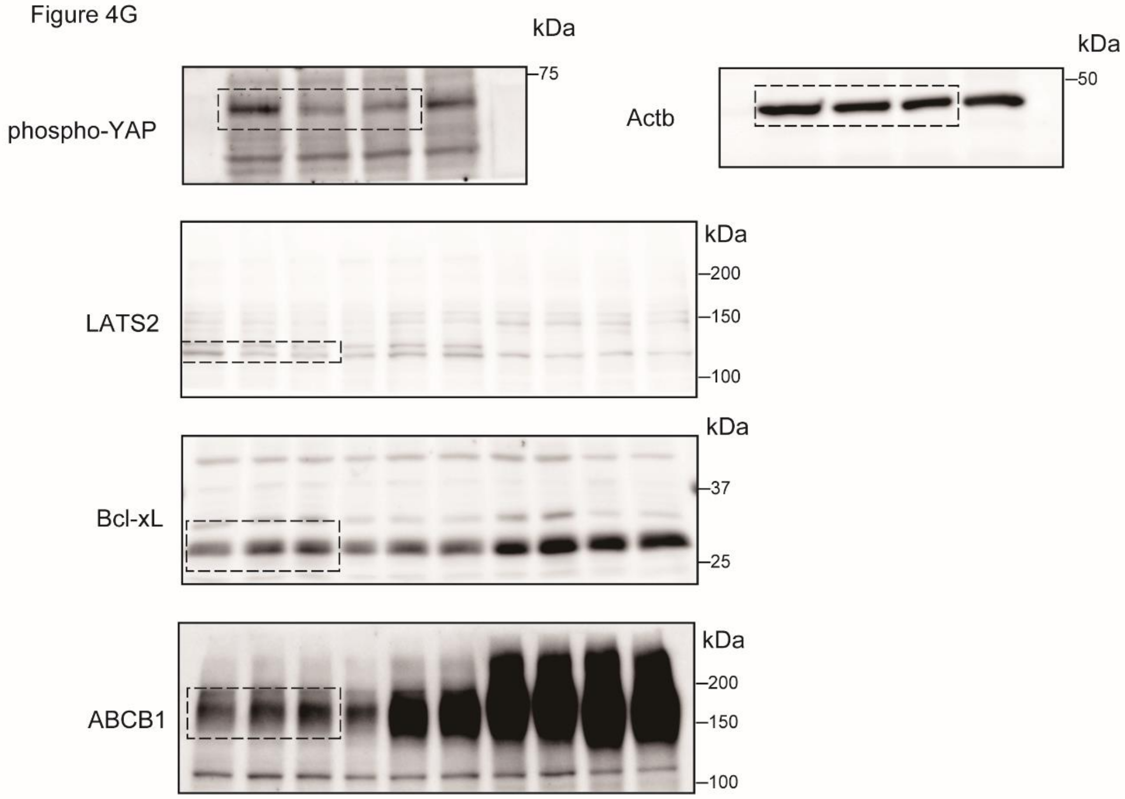

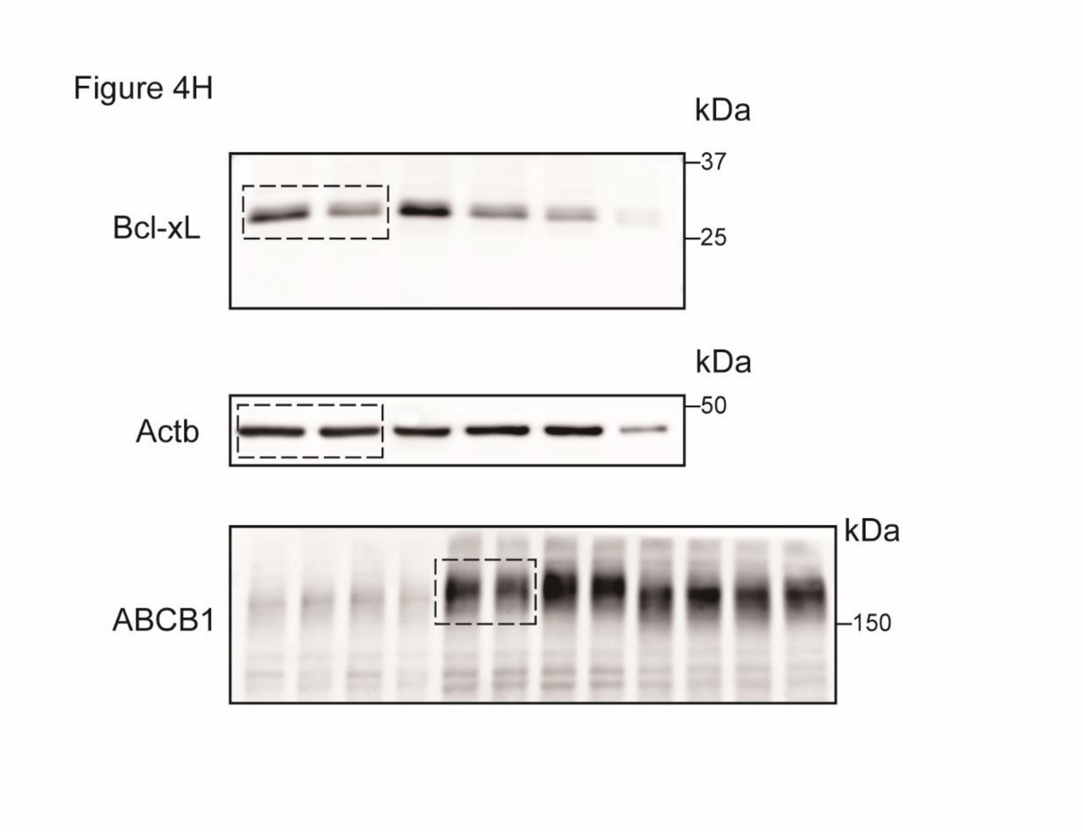

Figure S4. Original images of western blot with molecular weight markers.

© 2019 by the authors. Licensee MDPI, Basel, Switzerland. This article is an open access article distributed under the terms and conditions of the Creative Commons Attribution (CC BY) license (http://creativecommons.org/licenses/by/4.0/).
